# Paper Sludge Reuse in Lightweight Aggregates Manufacturing

**DOI:** 10.3390/ma9110876

**Published:** 2016-10-27

**Authors:** How-Ji Chen, Ying-Chih Hsueh, Ching-Fang Peng, Chao-Wei Tang

**Affiliations:** 1 Department of Civil Engineering, National Chung-Hsing University, No. 250, Kuo Kuang Road, Taichung 402, Taiwan; hojichen@nchu.edu.tw (H.-J.C.); iforsion@yahoo.com.tw (Y.-C.H.); cute1309@gmail.com (C.-F.P.); 2 Department of Civil Engineering & Geomatics, Cheng Shiu University, No. 840, Chengcing Rd., Niaosong District, Kaohsiung 83347, Taiwan

**Keywords:** paper sludge, lightweight aggregate, green building materials

## Abstract

The lightweight aggregates used by the civil engineering market are sintered at a high temperature, about 1200 °C. In times of high energy prices and regulation of carbon dioxide emissions, lightweight aggregate products of the high-temperature process in sales marketing are not readily accepted. This study developed a sintered-type paper sludge lightweight aggregate. In order to reduce energy consumption, substitution of some reservoir sediment clay in paper sludge substitutes is to be expected. The study used two types of paper sludge (green clay paper sludge and paper pulp sludge). The sintering temperature was reduced effectively as the green clay paper sludge was substituted for some of the reservoir sediment clay, and the optimum substitute ranges of green clay paper sludge were 10%–50%. The optimum substitute ranges of the paper pulp sludge were 10%–40%. Test results show that the properties of aggregates have a particle density of 0.66–1.69 g/cm^3^, a water absorption of 5%–30%, and a loss on ignition of 10%–43%. The loss on ignition of aggregate became greater with the increase in paper sludge content. This means that the calorific value provided by the paper sludge will increase as paper sludge content increases. Paper sludge can therefore be considered a good material to provide heat energy for sintering lightweight aggregate.

## 1. Introduction

Lightweight aggregates with lightness, durability, excellent thermal insulation, and high strength are suitable for building construction. They have been used in many countries as construction materials for many years [1,2]. In Taiwan, about 100,000 cubic meters of lightweight aggregate are used in the market per year, of which about 80% are imported from Mainland China. Since only a small amount of lightweight aggregate is produced in Taiwan, the cement and concrete products industry needs to import high cost aggregates. As a result, the industry has lost interest in using lightweight aggregates. This is also the major reason that the lightweight aggregate industry cannot expand quickly in Taiwan.

The lightweight aggregates currently used were sintered at a high temperature, about 1200 °C. In times of high energy prices and regulation of carbon dioxide emissions, the sale and marketing of the high-temperature processed products are quite difficult. To solve this dilemma, many methods have been proposed [3,4,5,6,7,8,9,10,11]. For instance, paper sludge has been generated after the settlement and dehydration processing of paper waste sludge. Essentially, paper sludge fibers are porous and organic, and so are uniquely lightweight and flammable. Paper sludge ash has low sintering activity at temperatures below 1200 °C [5]. It has been proved that paper sludge lightweight aggregates can be used to replace current high-temperature sintered products [12,13,14,15]. The paper industries report that about 0.3 million tons of paper sludge are generated per year in Taiwan. Since paper sludge is a refuse-derived fuel (RDF) in the lightweight aggregate manufacturing process, energy consumption can be reduced and a sustainable resource can be reused [16,17]. In addition, lightweight aggregates could be obtained with alternative low temperature processes, such as cold bonding pelletization [6]. A number of papers have confirmed that cold bonding pelletization can be used successfully in the production of aggregates [7,8,9,10]. In general, cold-bonded lightweight aggregates may have a higher specific gravity, as compared with sintered lightweight aggregates. 

In view of the above, a sintered type of paper sludge lightweight aggregate was developed in this study. The paper sludge lightweight aggregate produced has excellent lightweight and thermal insulation properties and possesses the characteristics of green building materials.

## 2. Experimental Procedure

### 2.1. Experimental Program

This article investigates the production of paper sludge lightweight aggregate. This type of aggregate is sintered from reservoir sediment clay mixed with paper sludge. The sintering procedure consists of the following three steps: (1) granulation; (2) drying; and (3) sintering. Sintering may be further divided into a preheating phase and a sintering phase [6,18,19]. In the preliminary trial and error study, paper sludge was mixed with reservoir sediment clay in a 10%–90% weight ratio. The trials were carried out in Chung Hsing University laboratory in Taichung, Taiwan. The initial selections of the temperature for the preheating and sintering phases were 350–700 °C and 1000–1250 °C, respectively. Various sintering times and temperatures for lightweight aggregates were observed based on the results of about 80 trials at 10%, 20%, 30%, 40%, and 50% weight ratios of paper sludge mixed with sediment clay. Temperatures of 400 °C and 600 °C for the preheating phase and 1125 °C and 1225 °C for the sintering phase were selected for subsequent experiments. An average value of three specimens was adopted for each test result.

### 2.2. Materials

**Figure 1:**
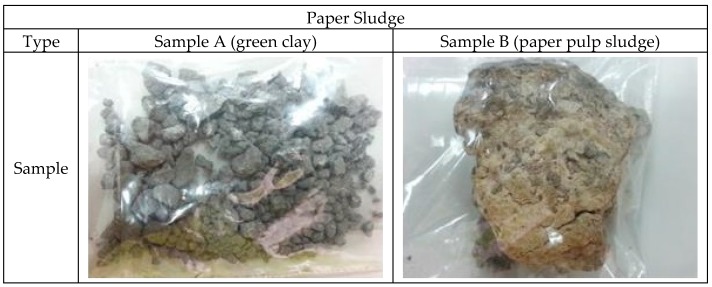
Paper sludge samples.

In this study, two types of paper sludge were tested, green clay paper sludge (secondary sludge), namely Sample A, and paper pulp sludge (primary sludge), namely Sample B. Green clay paper sludge was collected form the secondary processing of paper waste sludge, with a moisture content of approximately 12.5%. It can be observed in Figure 1 that the sample was nearly dry. Sample B is light yellow fibrous paper pulp sludge that was collected from the first processing of paper waste sludge, with a moisture content of approximately 131%. The sediment clay was collected from Shimen Reservoir in Taoyuan, Northern Taiwan. The particle size of reservoir sediment clay is quite small; the middle value is about 10 μm. The mixtures of paper sludge lightweight aggregates are listed in Table 1. Samples of the raw materials (i.e., paper sludge and sediment clay) were dried prior to use in the graining process. Subsequently, each sample was crushed and milled by a Leading-Coming-Air Type crushing machine or a Dry Ball mill. According to the designed proportions, the fine powders of the raw materials were thoroughly mixed to ensure good homogeneity. Then, a controlled amount of water (about 25%) was added to the mixture to produce a mix consistency that allowed for the formation of cylindrical pellets approximately 8 mm in diameter and 10 mm in length using a self-made extrusion granulator. Using the pressure extrusion process, the fibrous paper sludge was mixed with the sediment clay, and the granulation process was easily continued.

## 3. Results and Discussion

### 3.1. Chemical Composition of Paper Sludge and Sediment Clay

The chemical composition of the two types of paper sludge and the sediment clay is shown in Table 2, which is discussed as follows:

#### 3.1.1. SiO_2_, Al_2_O_3_, and Fe_2_O_3_

Silica (SiO_2_), alumina (Al_2_O_3_), and iron oxide (Fe_2_O_3_) are the main elements of the lightweight aggregates. The total amount of these chemical compounds in Samples A and B are 2.86% and 32.3%, respectively. Because of the low presence of these chemical compounds, the materials are not suitable for production of lightweight aggregate [20,21,22]. This is significantly noticeable in Sample A.

#### 3.1.2. Fe_2_O_3_, Na_2_O, K_2_O, CaO, MgO, and FeO

Fe_2_O_3_, sodium oxide (Na_2_O), potassium oxide (K_2_O), calcium oxide (CaO), magnesium oxide (MgO), and ferrous oxide (FeO) are known to be fluxing elements during the sintering process [20,21,22]. The presence of these compounds will affect the softening temperature of the material. As their presence increases, the sintering temperature decreases. With its high flux content (53.29%), it is expected that Sample A can decrease the sintering temperature. The flux content in Sample B is low (3.30%) relative to Sample A; Sample B cannot effectively reduce the sintering temperature.

#### 3.1.3. Mass Ratio of SiO_2_ and Al_2_O_3_ to Fluxing

Without considering the loss on ignition in the chemical composition, only the content of SiO_2_, Al_2_O_3_, and fluxing was calculated. The chemical composition ratio of the test materials is shown in Table 3. The mass ratio of (SiO_2_ + Al_2_O_3_)/Fluxing ranging between 3.5 and 10.2 is preferred for the expansion of lightweight aggregate. Table 3 shows that the corresponding values for Sample A and Sample B were 0.03 and 10.11, respectively. It can be seen that the mass ratio of Sample A did not appear within the preferred range. This indicates that the viscosity of the mixture will be reduced during the sintering process. The expanded gases may escape and result in a poor performance of foaming expansion [20,22].

#### 3.1.4. Three Phase Diagram of Chemical Compounds

**Figure 2:**
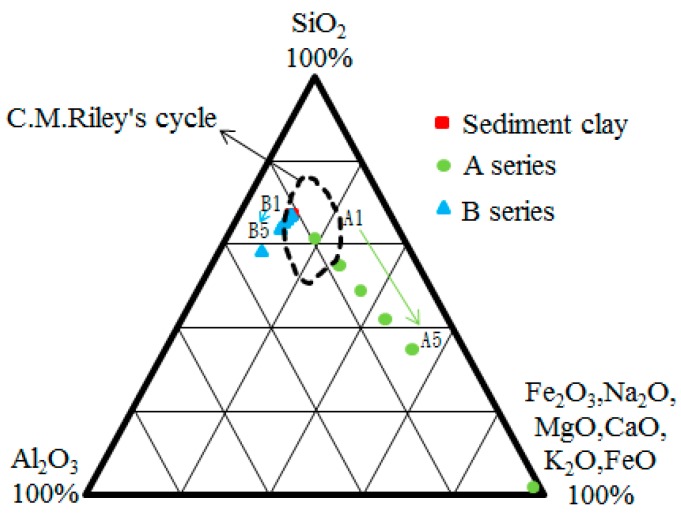
Ternary phase diagram of the chemical compositions of the test materials.

The chemical compounds in the sediment clay, Sample A, and Sample B are analyzed. The test results are shown in Table 3. The content of SiO_2_ was 66%, 2%, and 57%, of Al_2_O_3_ 23%, 1%, and 34%, and of flux 11%, 97%, and 9%, respectively. In the study, 10%, 20%, 30%, 40%, and 50% weight ratios of paper sludge were mixed with sediment clay. These chemical compound results of the mixtures are summarized in Table 3 and shown in the three phase diagram in Figure 2. Table 3 shows that the mass ratio of (SiO_2_ + Al_2_O_3_)/Fluxing for Sample A series is between 4.10 and 0.85; for the Sample B series, it is between 8.26 and 9.00. It can be seen that the mass ratios occurred within the preferred range (3.5–10.2), except the case of Samples A2, A3, A4, and A5. Compared with the suggested range of Riley’s chemical composition cycle [20], as shown in Figure 2, it was observed that only Samples A3, A4, and A5 are quite far away from the cycle; the other test group results fell within or close to the range. This result suggests that the mixture of paper sludge with sediment clay should be a potential raw material for lightweight aggregates.

### 3.2. Sintering of Paper Sludge Lightweight Aggregates

**Figure 3:**
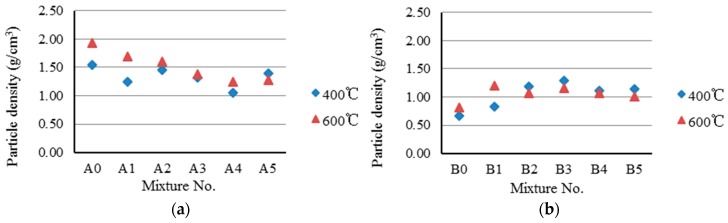
Particle density of paper sludge lightweight aggregates. (**a**) A series; (**b**) B series.

**Figure 4:**
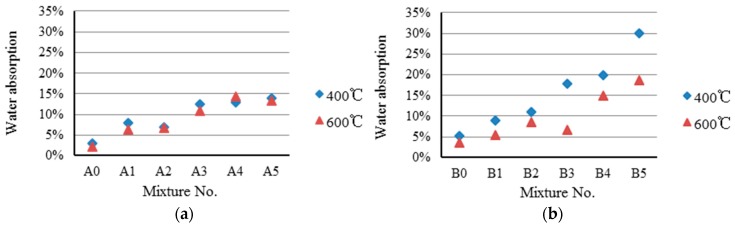
Water absorption of paper sludge lightweight aggregates. (**a**) A series; (**b**) B series.

Based on the preliminary trial results, the preheating temperature was set to 400 °C and 600 °C, as shown in Table 4. As can be seen in Table 4, the sintering temperature of the Sample A series (1125 °C) is lower than that of the Sample B series (1225 °C). The reason is that the test groups of the Sample A series possess high flux content, which decreases the sintering temperature. The oven-dried particle density and 24-h water absorption of paper sludge lightweight aggregates are summarized and presented in Figure 3 and Figure 4. Table 4 shows that the particle density range of lightweight aggregates for Sample A series is between 1.06 and 1.69 g/cm^3^; for Sample B, the series is between 0.83 and 1.30 g/cm^3^. In Table 4, it can also be seen that the particle densities of Sample A or Sample B series are less than 1.8 g/cm^3^, except for the experimental control group of A0. This meets the required primary property of specification recommended for lightweight aggregates. Figure 3a (Sample A series) shows that the particle density decreases as the paper sludge content increases. However, a similar trend is not seen in Figure 3b (Sample B series).

As shown in Figure 4, the 24-h water absorption values of Samples A series range from 6% to 14%, the water absorption of Samples B series between the values 5% and 30%. For both Samples A and B series, the values increase as the paper sludge content increases. Furthermore, the water absorption is generally higher when a lower preheating temperature (400 °C) is used. For structural concrete engineering applications, the water absorption of lightweight aggregates is usually less than 20%. The results indicate that the lightweight aggregates in this study meet with regulations, except for Sample B5.

**Figure 5:**
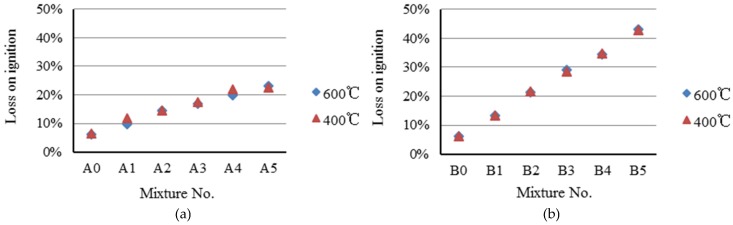
Loss on ignition of paper sludge lightweight aggregates. (**a**) A series; (**b**) B series.

The test results of loss on ignition are shown in Figure 5. It was observed that the Sample B series has higher loss on ignition values (13%–43%) than those of the Sample A series (10%–23%). The loss on ignition values of both Samples A and B series increase as paper sludge content increases. It was also found that no different effect between the preheating temperature of 400 °C and 600 °C is apparent. The greater the loss on ignition of the aggregates, which implies that more paper sludge was burned and converted into thermal energy, more pores will be produced in the aggregates, resulting in the particle density of lightweight aggregate decreasing. The test results, shown in Figure 3a (Sample A series), indicate that particle density decreases as paper sludge content increases. However, a similar trend is not seen in Figure 3b (Sample B series).

**Figure 6:**
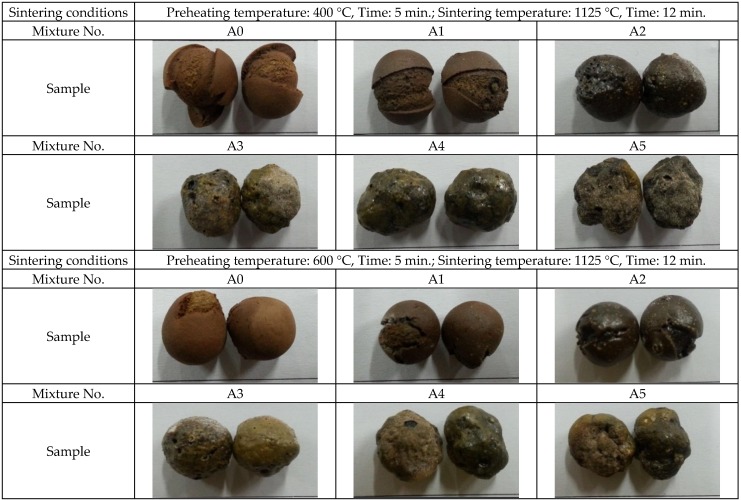
Appearance of paper sludge lightweight aggregate products (A series).

**Figure 7:**
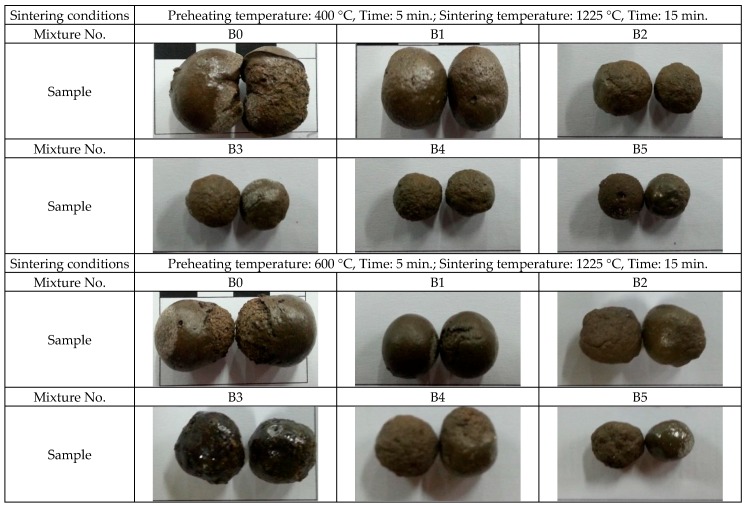
Appearance of paper sludge lightweight aggregate products (B series).

Referring to the sintered products of lightweight aggregate presented in Figure 6, it was found that Sample A series stay approximately the same size, and their expansion rates are close. Therefore, the loss on ignition increases as paper sludge content increases, which results in the reduction of particle mass and a decrease in particle density. On the other hand, Figure 7 shows that the particle size and expansion rate of the Sample B series decreases as the paper sludge content increases. The loss on ignition values is close to the content of paper sludge, which implies that most of the paper sludge was burned and converted into thermal energy, resulting in the reduction of particle mass. However, the expansion rate decreases (Figure 7) such that the Sample B series undergoes no significant change in its particle density. The particle density of lightweight aggregates for general structural concrete is 1.0–1.8 g/cm^3^; for non-structural concrete, it is less than 1.0 g/cm^3^. If the 24-h water absorption is less than 20%, it is suitable for structural concrete—otherwise for non-structural concrete. Based on the test results, the substitute ranges of green clay are 10%–50%, and the substitute ranges of the paper pulp sludge are 10%–40%. The properties of paper sludge lightweight aggregates meet the required primary property of specification recommended for lightweight aggregates. It is clear that the mixes of paper sludge tested in this study can be used to produce both structural and nonstructural concrete using lightweight aggregates.

## 4. Conclusions

Based on the above results and discussion, the following conclusions can be drawn:

1. Based on the chemical composition analysis of paper sludge, the paper sludge is not suitable for producing lightweight aggregate. The mixture of sediment clay with a weight ratio of 10%–50% paper sludge, however, should be a potential raw material for lightweight aggregates.
2. The sintering temperature can be effectively reduced with the high flux content in green clay series lightweight aggregates. On the other hand, the low flux content in paper pulp sludge series lightweight aggregates is ineffective.
3. Green clay lightweight aggregates stay in approximately the same size, and their expansion rates are close. The loss on ignition increases as green clay content increases, resulting in the reduction in particle mass and a decrease in particle density. On the other hand, the particle size and expansion rate of paper pulp sludge lightweight aggregates decreases as the paper pulp sludge content increases. Even though the loss on ignition values increases, resulting in the reduction of particle mass, the paper pulp sludge lightweight aggregates is without significant change in its particle density. The reason is that particle sizes and expansion rates decrease as paper pulp sludge content increases.
4. The particle density range of green clay lightweight aggregates is between 1.06 and 1.69; for paper pulp sludge lightweight aggregates, it is between 0.83 and 1.30. This meets the required primary property of recommended specifications of lightweight aggregates.
5. The 24-h water absorption values of green clay lightweight aggregate range from 6% to 14%, and the water absorption of paper pulp sludge lightweight aggregate range between the values of 5% and 30%. The values increase as paper sludge content increases. For structural concrete engineering applications, the water absorption of lightweight aggregates is usually less than 20%. The results indicate that the lightweight aggregates in this study comply with regulations, except the case of Sample B5.
6. Both the green clay and paper pulp sludge can provide good thermal energy under the sintering process and can be used as refuse-derived fuel, as well as raw materials to produce lightweight aggregates.

## Figures and Tables

**Table 1:** Mixtures of paper sludge lightweight aggregates (weight ratios).

Mixture No.	A0, B0	A1, B1	A2, B2	A3, B3	A4, B4	A5, B5
Sediment clay	100	90	80	70	60	50
Paper sludge	0	10	20	30	40	50

**Table 2:** Chemical composition of the test materials.

Sludge Type	Chemical Compounds (%)											
	SiO_2_	Al_2_O_3_	Fe_2_O_3_	Na_2_O	MgO	CaO	K_2_O	SO_3_	Others	Organisms	LOI	Total
Sediment clay	59.31	19.97	6.53	0.01	2.02	1.41	0.08	0.07	–	2.90	7.7	100
Sample A	1.25	0.31	1.30	1.43	2.73	47.66	0.17	1.62	1.08	–	42.5	100
Sample B	20.10	11.90	0.30	–	2.20	0.80	–	–	2.50	–	62.2	100
Proper proportion *	53–79	12–26	8–24					–	–	–	–	–

Note: * = Expandable region of the ternary diagram produced by Riley [20]; LOI = Loss on ignition.

**Table 3:** Chemical composition ratio of the test materials.

**Chemical Composition**	**A- Mixture No.**						
	**Sample A**	**Sediment Clay (A0)**	**A1**	**A2**	**A3**	**A4**	**A5**
SiO_2_	2	66	60	53	47	40	34
Al_2_O_3_	1	23	21	19	16	14	12
Fluxing	97	11	20	28	37	45	54
Total	100	100	100	100	100	100	100
(SiO_2_ + Al_2_O_3_)/Fluxing	0.03	8.09	4.10	2.55	1.72	1.20	0.85
**Chemical Composition**	**B- Mixture No.**						
	**Sample B**	**Sediment Clay (B0)**	**B1**	**B2**	**B3**	**B4**	**B5**
SiO_2_	57	66	65	64	63	62	62
Al_2_O_3_	34	23	24	25	26	27	29
Fluxing	9	11	11	11	10	10	10
Total	100	100	100	100	100	100	100
(SiO_2_ + Al_2_O_3_)/Fluxing	10.11	8.09	8.26	8.43	8.62	8.80	9.00

**Table 4:** Production results of paper sludge lightweight aggregates.

Mixture No.	Preheating Conditions		Sintering Conditions		Loss On Ignition (%)	Water Absorption (%)	Oven Dry Particle Density (G/Cm^3^)
	Temperature (°C)	Time (min)	Temperature (°C)	Time (min)			
A0	400	5	1125	12	6	3	1.55
A1					12	8	1.24
A2					14	7	1.45
A3					17	13	1.33
A4					22	13	1.06
A5					23	14	1.40
A0	600	5	1125	12	6	2	1.93
A1					10	6	1.69
A2					14	7	1.61
A3					17	11	1.39
A4					20	14	1.24
A5					23	13	1.28
B0	400	5	1225	15	6	5	0.66
B1					13	9	0.83
B2					22	11	1.18
B3					28	18	1.29
B4					35	20	1.11
B5					43	30	1.14
B0	600	5	1225	15	6	4	0.86
B1					13	5	1.19
B2					21	8	1.06
B3					29	6	1.30
B4					35	15	0.97
B5					43	14	1.15
